# Assessing the quality of shared decision making for elective orthopedic surgery across a large healthcare system: cross-sectional survey study

**DOI:** 10.1186/s12891-021-04853-x

**Published:** 2021-11-19

**Authors:** K. D. Valentine, Tom Cha, John C. Giardina, Felisha Marques, Steven J. Atlas, Hany Bedair, Antonia F. Chen, Terence Doorly, James Kang, Lauren Leavitt, Adam Licurse, Todd O’Brien, Thomas Sequist, Karen Sepucha

**Affiliations:** 1 Massachusetts General Hospital (MGH), 100 Cambridge Street, 16th floor, Boston, MA 02114 USA; 2 Harvard Medical School (HMS), Boston, MA USA; 3 Harvard T.H. Chan School of Public Health, Boston, MA USA; 4 North Shore Medical Center, MA Salem, USA; 5 Newton Wellesley Hospital, MA Newton, USA; 6 Brigham and Women’s Hospital (BWH), MA Boston, USA; 7 Department of Quality and Patient Experience, Mass General Brigham Health System, Boston, MA USA

**Keywords:** Shared decision making, Quality measurement, Osteoarthritis, Total joint replacement, Spine surgery

## Abstract

**Background:**

Clinical guidelines recommend engaging patients in shared decision making for common orthopedic procedures; however, limited work has assessed what is occurring in practice. This study assessed the quality of shared decision making for elective hip and knee replacement and spine surgery at four network-affiliated hospitals.

**Methods:**

A cross-sectional sample of 875 adult patients undergoing total hip or knee joint replacement (TJR) for osteoarthritis or spine surgery for lumbar herniated disc or lumbar spinal stenosis was selected. Patients were mailed a survey including measures of Shared Decision Making (SDMP scale) and Informed, Patient-Centered (IPC) decisions. We examined decision-making across sites, surgeons, and conditions, and whether the decision-making measures were associated with better health outcomes. Analyses were adjusted for clustering of patients within surgeons.

**Results:**

Six hundred forty-six surveys (74% response rate) were returned with sufficient responses for analysis. Patients who had TJR reported lower SDMP scores than patients who had spine surgery (2.2 vs. 2.8; *p* < 0.001). Patients who had TJR were more likely to make IPC decisions (OA = 70%, Spine = 41%; *p* < 0.001). SDMP and IPC scores varied widely across surgeons, but the site was not predictive of SDMP scores or IPC decisions (all *p* > 0.09). Higher SDMP scores and IPC decisions were associated with larger improvements in global health outcomes for patients who had TJR, but not patients who had spine surgery.

**Conclusions:**

Measures of shared decision making and decision quality varied among patients undergoing common elective orthopedic procedures. Routine measurement of shared decision making provides insight into areas of strength across these different orthopedic conditions as well as areas in need of improvement.

**Supplementary Information:**

The online version contains supplementary material available at 10.1186/s12891-021-04853-x.

## Background

Shared decision making (SDM) is recommended by clinical guidelines for many elective orthopedic surgeries as a way of ensuring that patients are well-informed about their treatment options, including non-surgical options, and that their final decision aligns with their informed preferences and goals [1–3]. Using SDM-based interventions such decision aids have been shown to improve patients’ decision-making process and, in some cases, reduce surgery rates [4–6]. As a result, groups are now advocating for SDM and decision aid use as part of total joint replacement bundles [7–9]. In order to systematically improve SDM across health systems, however, there is a need for practical, reliable, and valid quality measures for the level of SDM for patient-provider interactions in routine care. Only once such measures are developed and routinely implemented in care can the effect of efforts to promote SDM be accurately evaluated.

In the context of elective orthopedic surgery, the National Quality Forum has endorsed two patient-reported measures of SDM: the SDM Process (SDMP) scale and the Informed, Patient-Centered (IPC) measure [10]. Studies have found that SDMP scores and IPC decisions are related to improved overall and disease-specific health outcomes, higher patient satisfaction, and decreased decisional regret in common orthopedic surgical decisions [11, 12]. To date, there are no published studies reporting results for these measures in routine elective orthopedic care (i.e., outside of a trial for a specific intervention). A better understanding of the quality of the decision-making process for routine elective orthopedic surgeries would help researchers and health systems identify best practices as well as opportunities for improvement.

The goal of this study was to measure SDM within a large healthcare network using National Quality Forum-endorsed performance measures for four common elective orthopedic surgeries: hip and knee total joint replacement (TJR) for osteoarthritis or spine surgery for lumbar herniated disc or lumbar spinal stenosis. We examined [1] the variation in IPC decision rates and SDMP scores across surgeons, practice sites, and surgery type and [2] whether patient reported outcomes are associated with IPC decision rates and SDMP scores.

## Methods

### Sample

We conducted a cross-sectional survey of a randomly selected subset of patients who had TJR or spine surgery at four hospitals (two academic medical centers and two community hospitals) that are part of a large healthcare system. Thirty-six surgeons across the four sites were included; 22 surgeons performed TJR, 15 surgeons performed spine surgery, and 1 surgeon performed both types of surgery. The participating surgeons were identified by the department chiefs at each hospital.

Patients were eligible to participate in the study if they had undergone elective hip or knee TJR to treat osteoarthritis (OA) or elective spine surgery to treat lumbar herniated disc (HD) or spinal stenosis (SS) by a participating surgeon between January 2018 and June 2018 (detailed inclusion criteria described in eTable1), and had pre-operatively completed the patient-reported outcome measures (PROMs) described in the “Outcomes, measures, and instruments” section below. (An exception was made for HD patients, who were surveyed regardless of pre-operative PROMs completion, because of the small size of that group). Spine patients were surveyed 4–26 weeks post-operatively and total joint replacement patients 12–26 weeks post operatively. The time window was set to survey patients outside the immediate recovery period, but not too long after the procedure. Study staff identified patients using a validated algorithm to search electronic health records for relevant ICD and CPT codes, and confirmed eligibility with a limited chart review [13]. Eligible patients who had undergone TJR were then randomly selected to be included in the study, stratified by surgeon, site, and condition to match the sample size target described in the “Statistical Analyses” subsection below; all eligible patients who had undergone spine surgery were selected for inclusion in the study because of the relatively smaller size of those groups.

### Survey procedure

Selected eligible patients were mailed a survey packet with a $2 incentive. A modified Dillman approach with reminder calls and follow-up mailings was used to increase the response rate [14].

All participant screening and enrollment data was tracked using a Microsoft Access database, and survey responses were entered into Research Electronic Data Capture software, an online database program. The study was approved by the Institutional Review Board at the Mass General Brigham HealthCare System (protocol 2005P002282).

### Outcomes, measures, and instruments

The Hip OA, Knee OA, HD, and SS Decision Quality Instruments (DQI) were used to determine whether or not a decision to have surgery was informed and patient-centered (IPC). Each survey includes (a) five multiple-choice knowledge questions and (b) one treatment preference question (i.e., prefer surgery, nonsurgical treatment, or not sure). The DQIs have been shown to have strong psychometric properties (such as test-retest reliability, validity, and sensitivity), as well as clinical sensibility (such as acceptability and feasibility) [15, 16]. A patient was coded as having an IPC decision if they answered at least 60% (for patients who had TJR) or 40% (for the patients who had spine surgery) of the knowledge questions correctly and indicated a clear preference for surgery. Knowledge thresholds were based on recommendations from the DQI scoring guides [15, 16]. As all patients in this sample received surgery, a desire for non-surgical treatment or being unsure would indicate a lack of clear preference. Patients missing responses to 3 or more of the knowledge questions and/or the treatment preference question were excluded from IPC analyses.

The SDM Process (SDMP) survey includes four questions asking whether or not the surgeon discussed: (1) non-surgical options, (2) benefits of surgery, (3) drawbacks of surgery, and (4) patient’s preference. Respondents with one or more missing items were excluded. SDMP total scores range from 0 to 4, with higher scores indicating higher SDM. The survey has strong evidence of both internal consistency and retest reliability (Cronbach’s α = 0.78 and retest intraclass correlation = 0.64) [11] and construct validity for surgical decisions [17–19].

All patients completed PROMIS Global Health Scales (including Mental Health and Physical Health scales) and the Physical Function scale. The Mental Health and Physical Health scales each include 4 items and converted scores range from 16.2–67.7, with higher values indicating better mental or physical health [20, 21]. PROMIS Physical Function questionnaire (Short Form 10a) is a 10-item survey. Converted scores range from 13.5–61.9, with higher values indicating greater capability to perform physical activities [22].

Demographic information (age, sex, ethnicity, site), type of procedure, date of procedure, visit note, specialist, prescription of a decision aid, and completed pre-operative PROMIS scores were collected from the electronic medical record. Ethnicity data was not available for 37 patients, so race/ethnicity was coded based on their race.

### Statistical analyses

First, we examined response rates and compared responders to non-responders using simultaneous multivariable logistic regression predicting response using hospital, surgery (i.e. TRJ or spine surgery), sex, age, race (white v. not), and ethnicity (Hispanic v. not). Then, we calculated the two performance measures, SDMP scores and rates of IPC decisions. We calculated scores and rates for the entire sample, each hospital, and individual surgeons with at least 15 patient responses. Sensitivity analyses were performed to identify if differences in SDMP and IPC decisions were present by condition (i.e. hip vs. knee osteoarthrosis and lumbar herniated disc vs. lumbar spinal stenosis) and to examine whether there were differences due to timing of survey. There were no differences in decision making outcomes between conditions so the four conditions were collapsed into two surgical conditions—TJR (including both hip and knee osteoarthrosis), and spine surgery (including lumbar herniated disc and lumbar spinal stenosis). Sensitivity analyses also found no relationship between the timing of the survey and decision making outcomes. We examined two questions: (1) is there variation in rates of IPC decisions and SDMP scores across hospitals, surgeon, and surgery type; and (2) what is the association between the change in pre- to post-operative PROMs and rates of IPC decisions and SDMP scores?

For question 1, multivariable regressions implementing generalized estimating equations (GEE) were used to correct for correlated error due to patients being nested within physicians. Logistic regression models predicted whether or not a patient made an IPC decision using the following variables: the hospital where the participant underwent surgery, the surgery (TJR or spine surgery), and patient age, race (white/non-Hispanic or other), and sex. Additionally, participant SDMP scores were predicted using an analogous simultaneous multivariable linear regression. Descriptive information was also used to summarize the individual items of the SDMP scale.

For question 2, differences in PROMs (PROMIS physical health, physical function, and mental health scores) pre- and post-surgery were analyzed with dependent t-tests and Cohen’s ds. Changes in PROMs were coded such that positive values indicated an improvement in that area following surgery. This score was standardized by dividing by the standard deviation of the pre-surgery scores. Only patients with complete pre- and post-surgery PROMs were included in these analyses. Six multiple linear regression models were analyzed using GEE: standardized changes in each PROM was predicted by whether the patient made an IPC decision and their SDMP score, as well as their age, sex, and race (white/non-Hispanic vs. other). A priori α = 0.05, all tests were 2-sided, and all analyses were conducted in RStudio version 1.1.447 using R version 19.6.0 [23, 24]. All code for graphs (ggplot2 package), t-tests (psych package), GEE models (geepack package), and Cohen’s d (MOTE package) can be found in the online supplements [25–30].

The a priori sample size target was 600 patients and was set to have 80% power to detect a 16% difference in IPC rates (e.g. from 20 to 36%) and a difference of 0.36SD in SDMP scores when comparing between sites or conditions. This target assumed 48 surgeons and an intraclass correlation coefficient of 0.005, resulting in an effective sample size of 486, or about 122 per site or per topic.

## Results

### Patient sample

**Fig. 1:**
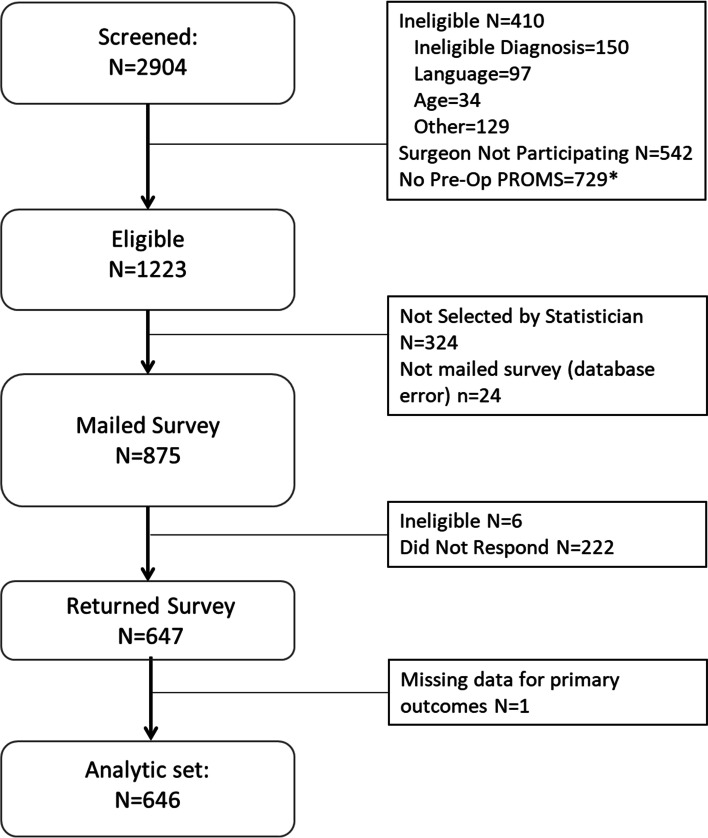
Recruitment Diagram. Note: Pre-Op PROMS = pre-operative patient reported outcomes completed. *Some herniated disc patients without Pre-Op PROMs were included to increase sample size for that condition

We received 647/875 surveys (74% response rate) and Fig. 1 shows the sample recruitment. One respondent did not complete the primary outcome measures and was removed from analysis, resulting in 646 observations. Response rates did not vary by hospital, condition, patient sex, race, or ethnicity (all *p* > 0.09). However, age was a significant predictor of responding; as age increased, patients were more likely to respond (OR = 1.03, 95% CI (1.02, 1.05), *p* < 0.001, model Pseudo R^2^ = 0.43). Age was included in all models to adjust for potential bias.

Median time between surgery and survey completion was 155 days (inter-quartile range = 30 days) and was similar across surgery type. Overall, 209 patients had hip replacement, 196 had knee replacement, 165 had surgery for SS, and 76 had surgery for HD. The patient sample was female (52%), white, non-Hispanic (93%), had an average age of 65, and 15% had been ordered a decision aid prior to their surgery (see Table 1).

**Table 1:** Patient Characteristics Across Hospitals

Variable	Hospital 1 (*n* = 212)	Hospital 2 (*n* = 212)	Hospital 3 (*n* = 85)	Hospital 4 (*n* = 137)	Overall (*n* = 646)
Age M (SD)	66 (11)	63 (12)	64 (11)	65 (11)	65 (11)
White, non Hispanic	91%	92%	95%	96%	93%
Female	56%	54%	44%	47%	52%
TJR surgery (vs Spine)	67%	63%	36%	72%	63%
Number of Surgeons^a^	15	14	6	9	36
Prescribed decision aid	9%	16%	16%	24%	15%

*M* Mean, *SD* Standard deviation, *TJR* Total joint replacement.

^a^7 surgeons operated at more than one hospital, 1 surgeon operated on hip, knee and spine patients

### Decision quality across hospital

Across all patients, the average SDMP score was 2.4 (SD = 1.1) on a scale from 0 to 4 and 60% (372/625) met both criteria for IPC decisions. The mean knowledge score was 59% (SD = 28%) and 78% (498/640) of patients indicated a clear preference for surgery. Table 2 shows the results by condition and hospital.

**Table 2:** Decision-making Outcomes Across Hospitals and Conditions

		Hospital 1	Hospital 2	Hospital 3	Hospital 4	Overall
	Sample Size	TJR = 141 Spine = 71	TJR = 134 Spine = 78	TJR = 31 Spine = 54	TJR = 99 Spine = 38	TJR = 405 Spine = 241
Shared Decision Making Process M (SD)	TJR	2.2 (1.1)	2.1 (1.1)	2.2 (1.1)	2.3 (1.1)	2.2 (1.1)
	Spine	2.8 (1.0)	2.9 (1.1)	2.6 (1.0)	2.8 (1.0)	2.8 (1.0)
% Informed^a^	TJR	78%	84%	67%	92%	83%
	Spine	60%	56%	64%	63%	60%
% Preferred Surgery	TJR	86%	82%	87%	84%	84%
	Spine	66%	68%	70%	63%	67%
Informed, Patient-Centered rate	TJR	68%	70%	62%	77%	70%
	Spine	40%	37%	46%	45%	41%

*Note*: A total of 7 patients did not have shared decision making process scores, 6 patients did not have a treatment preference, 15 patients did not have a knowledge score, and 21 patients did not have an informed, patient-centered score

*TJR* Total joint replacement, *M* Mean, *SD* Standard deviation, *SDM* Shared Decision Making; ^a^patients were considered “informed” if they answered at least 60% (for patients who had TJR) or 40% (for patients who had spine surgery) of the knowledge questions correctly.

### Decision quality across surgeon

**Fig. 2:**
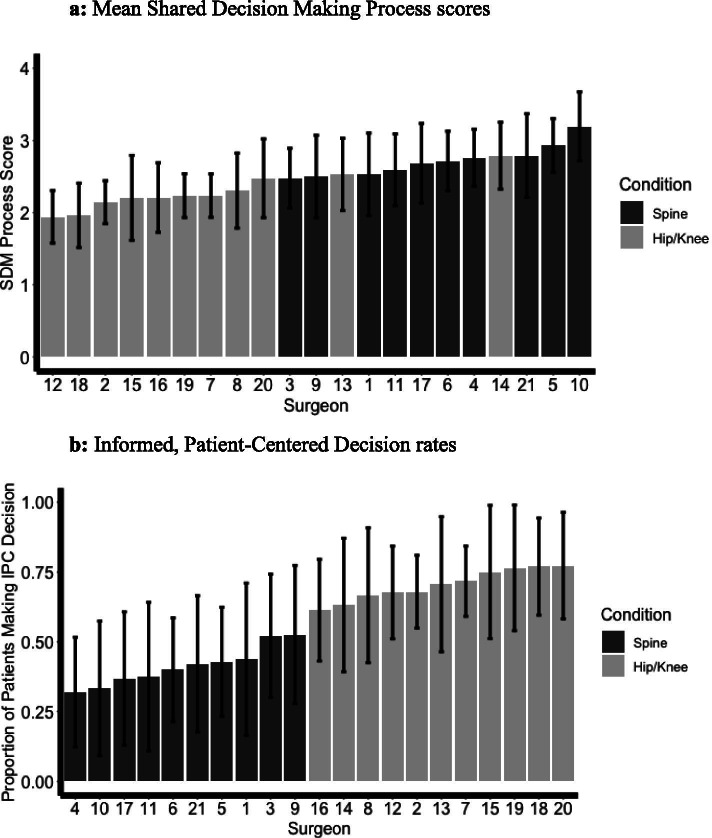
Shared Decision Making Process scores and Informed, Patient-Centered Decisions rates by surgeon. **a** Mean Shared Decision Making Process scores. **b** Informed, Patient-Centered Decision rates

Twenty-two surgeons had responses from 15 or more patients. Across this group, average SDMP scores ranged from 1.9 to 2.8 for hip and knee surgeons and from 2.5 to 3.2 for spine surgeons (see Fig. 2a). The mean IPC rates ranged from 63 to 81% for hip and knee surgeons and from 32 to 55% for spine surgeons (see Fig. 2b).

### Decision quality across surgery type

Regression analysis indicated that patients who had TJR reported lower SDMP scores than patients who had spine surgery (adjusted mean difference − 0.55, *p* < 0.001). The hospital where the surgery was performed, patient sex, and race/ethnicity were not predictive of SDMP scores (*p*s > 0.06, model *R*^*2*^ = 0.07).

Table 3 details item-level differences between patients who had TJR and patients who had spine surgery. While most patients who had TJR reported that surgeons talked about the benefits of TJR surgery and asked for their preferences, less than half reported a meaningful discussion of non-surgical options or downsides of surgery. The experience of patients who had spine surgery in this sample was better, as the majority reported that surgeons asked for their preference, discussed reasons to have surgery, non-surgical options and downsides of surgery.

**Table 3:** SDM Process Scale items by surgery

		Spine N (%)	TJR N (%)
Pros	A lot	146 (61)	199 (50)
	Some	73 (30)	145 (36)
	A little	14 (6)	47 (12)
	Not at all	6 (3)	9 (2)
Cons	A lot	67 (28)	49 (12)
	Some	86 (36)	113 (28)
	A little	35 (15)	99 (25)
	Not at all	51 (21)	139 (35)
Options	Yes	170 (71)	173 (43)
	No	69 (29)	227 (57)
Preferences	Yes	202 (85)	333 (83)
	No	37 (15)	67 (17)

Conversely, patients who had TJR were more likely to make IPC decisions than patients who had spine surgery (*b* = 1.31, *p* < 0.001). White, non-Hispanic patients (*b* = 0.53, *p* = 0.048) were also more likely to make IPC decisions. The hospital where the surgery was performed, patient age, and sex were not predictive of IPC decisions (*ps* > 0.056, model *R*^*2*^ = 0.10).

### Decision making measures and patient-reported outcomes

Table 4 contains the pre- and post-operative PROMs scores. Models were stratified by condition and results are discussed below. Full model results are available in Table 5.

**Table 4:** Pre and Post Surgery Patient Reported Outcome Scores

		N	Pre Mean (SD)	Post Mean (SD)	Mean Change	*P*	d
PROMIS Mental Health	TJR	351	51.1 (8.98)	54.8 (7.98)	3.63	<.001	0.43
	Spine	224	46.1 (8.90)	51.7 (9.51)	5.64	< 0.001	0.61
PROMIS Physical Health	TJR	348	41.7 (7.33)	50.4 (8.67)	8.65	< 0.001	1.08
	Spine	220	37.6 (7.10)	47.1 (8.62)	9.52	< 0.001	1.21
PROMIS Physical Function	TJR	347	37.3 (5.14)	45.6 (8.03)	8.31	< 0.001	1.23
	Spine	223	34.9 (5.14)	43.3 (8.80)	8.41	< 0.001	1.17

*Note*: *SD* Standard deviation, *TJR* Total joint replacement

**Table 5:** Estimates from models predicting standardized PROMIS change scores

		TJR		Spine	
		b (se)	*P*	b (se)	*P*
Mental Health	Intercept	1.69 (0.61)	0.01	1.72 (0.84)	0.04
	IPC decision	0.09 (0.14)	0.53	−0.09 (0.18)	0.63
	SDM Process	**0.16 (0.07)**	**0.02**	−0.02 (0.12)	0.86
	Age	**−0.03 (0.01)**	**0.00**	−0.01 (0.01)	0.26
	Sex (male)	**−0.55 (0.18)**	**0.00**	−0.03 (0.29)	0.92
	Race (white)	**0.90 (0.23)**	**0.00**	0.24 (0.31)	0.43
Physical Health	Intercept	4.46 (0.95)	0.00	4.07 (1.18)	0.00
	IPC decision	**0.95** (**0.20**)	**0.00**	−0.30 (0.44)	0.50
	SDM Process	**0.25** (**0.11**)	**0.02**	−0.14 (0.20)	0.48
	Age	**−0.04** (**0.01**)	**0.00**	**−0.02** (**0.01**)	**0.03**
	Sex (male)	−0.17 (0.33)	0.60	0.25 (0.35)	0.48
	Race (white)	0.49 (0.54)	0.37	**1.52** (**0.57**)	**0.01**
Physical Function	Intercept	2.77 (0.93)	0.00	4.89 (0.80)	0.00
	IPC decision	**0.51** (**0.19**)	**0.01**	**−0.62** (**0.26**)	**0.02**
	SDM Process	0.13 (0.08)	0.08	−0.20 (0.18)	0.26
	Age	**−0.03** (**0.01**)	**0.00**	**−0.05** (**0.01**)	**0.00**
	Sex (male)	**0.42** (**0.17**)	**0.02**	0.36 (0.22)	0.10
	Race (white)	0.66 (0.45)	0.14	**1.08** (**0.42**)	**0.01**

*Note*: Values that are bolded indicate they were significant predictors in their model. *TJR* Total joint replacement, *SDM* Shared decision making, *IPC* Informed, patient-centered decision

On average, patients reported higher PROMIS mental health scores after surgery (see Table 5). For patients who had TJR, greater improvement was associated with higher SDMP scores,  being younger, being white, and being female. IPC was not related to greater improvements in mental health for patients who had TJR. No predictors were associated with improvements in mental health for patients who had spine surgery (TJR model R^2^ = 0.09, Spine model R^2^ = 0.01).

On average, patients reported greater PROMIS physical health after surgery (see Table 5). For patients who had TJR, greater improvement in standardized change scores was associated with making an IPC decision, having higher SDMP scores, and being younger; while for patients who had spine surgery, being white and being younger was related to improved physical health. No other variables were related to improved physical health (TJR model R^2^ = 0.06, Spine model R^2^ = 0.04).

On average, patients reported greater PROMIS physical function after surgery (see Table 5). For patients who had TJR, greater improvement in standardized change scores was associated with making an IPC decision, being younger, and being male. However, for patients who had spine surgery, not making an IPC decision, being younger, and being white were associated with greater improvement in physical function. No other variables were related to improved physical function (TJR model *R*^*2*^ = 0.07, Spine model *R*^*2*^ = 0.12).

## Discussion

This study examined the quality of surgical decision making for elective hip and knee total joint replacement and lumbar spine degenerative disorders as part of usual care in a large health care system using two National Quality Forum-endorsed measures. Patients who had spine surgery reported high SDMP scores, consistently better than the hip and knee replacement patients across hospitals and surgeons. In contrast, the majority of patients who had TJR (70%) were informed and had a clear preference for surgery, meeting the criteria for IPC decisions. Less than half of the patients who had spine surgery (41%) were both well-informed and had clear preference for surgery. Scores on the measures did not vary significantly across hospitals, though there was considerable variability across surgeons. Both SDMP scores and IPC decisions were associated with small improvements in PROMIS Global Health scores for patients who had TJR in this sample. For patients who had spine surgery, the IPC rates and SDMP scores did not have a significant association with PROMIS Global Health scores; however, IPC rates were associated with a small decrease in PROMIS Physical Function scores.

Shared decision making involves the discussion of options, benefits and harms, and the exploration of patients’ preferences for treatment. While most TJR patients reported that surgeons talked about the benefits and asked for their preferences, less than half reported discussion of non-surgical options or downsides. Audiotaped analyses of orthopedic surgeon consultations have documented similar deficits in informed decision making, with discussion of both pros and cons and elicitation of patients’ preferences occurring less than half the time [31]. The experience of spine patients in this sample was better, as the majority reported that surgeons asked for their preference, and discussed reasons to have surgery, downsides of surgery, and non-surgical options. A national study of medical decisions also found that spine patients reported the highest SDM scores compared to other types of decisions [32]. Nearly all spine surgeons at these sites had higher scores than the TJR surgeons, but within each discipline, the highest scoring surgeon was almost a full standard deviation above the lowest, indicating room for improvement. Specifically, training in shared decision making and communication skills may be important for TJR surgeons.

The IPC findings confirm that patients who had TJR were knowledgeable of the benefits and risks of the procedure and the majority reported a clear preference for surgery. However, it’s important to remember that less than half of patients who had TJR discussed non-surgical options. Patients who had spine surgery had lower rates of IPC decisions, and somewhat surprisingly, only 63–70% reported a clear preference for surgery. Together, these results highlight challenges for spine surgery decision making. For example, determining the cause of back pain and sciatica can be difficult and explaining these conditions to patients can be challenging [33]. Further, spine surgery is not as effective as TJR which may result in patients being less sure about the choice [34]. The fairly low rate of IPC decisions suggests that the visits with spine surgeons may not be enough to overcome the inherent difficulties in the decision-making process for spine surgery and underscores the need for additional decision support. Specifically, interventions to improve patients’ knowledge, such as patient decision aids, may be particularly beneficial for patients considering spine surgery [35].

The main purpose of these surgeries is to improve patient reported outcomes. In general, patients who had spine surgery in this sample had lower PROMIS scores pre-operatively compared to the patients who had TJR. Both groups reported significant improvements in outcomes after surgery, and the magnitude of improvement in physical health and function was similar for patients who had TJR and spine surgery. The improvements in mental health were larger for patients who had spine surgery compared to patients who had TJR, a finding that has been seen in the literature [36]. For patients who had TJR, SDMP scores, IPC rates, patient sex (being male), younger age, and race (White) were also associated with improvements in health outcomes. Other studies have also found differences by sex, with females being more likely to receive TJR later, and to have slightly worse outcomes compared to males [37, 38]. For spine surgery, the unexpected finding that not making an IPC decision was associated with small improvements in physical function may be a result of the sampling procedure. A previously published single site study found a strong, positive relationship between IPC rates and outcomes for patients who had spine surgery, although that study enrolled patients prospectively and included patients who pursued both surgical and non-surgical treatment [12]. These findings are crucial to our understanding of how decision quality in routine elective orthopedic care relates to health outcomes as there is little evidence of these relationships outside of intervention trials.

Engaging and informing patients can be difficult given multiple competing priorities and short time allocated for patients visits. Decision aids have been shown to increase knowledge and the percentage of patients who receive their preferred treatment [35]. Although decision aids were available to these surgeons, use in routine care was quite low. If the decision making metrics can be collected and used to provide clinicians with feedback on their performance, this may encourage clinicians to adopt decision aids or other evidence-based interventions to improve the quality of decisions. Prior work suggests that the use of these types of audit and feedback loops can benefit clinicians by identifying areas of strengths, as well as limitations [39]. If this feedback can be integrated and clinician skills improved, this could lead to better decision quality for patients.

There are several limitations of this study. First, patients were surveyed a median of 22.1 weeks after undergoing surgery and may be subject to recall bias. One might expect that knowledge scores would decline over time, and that patients would be more likely to indicate a clear preference for the procedure after it happened. However, in this sample, no relationships between these decision making outcomes and time since surgery were found. Second, given the differences between TJR and spine surgery, we stratified analyses by condition resulting in smaller samples and reduced statistical power for some analyses. Third, the patient sample at these sites had limited racial and ethnic diversity limiting generalizability. Finally, data regarding access to rehabilitation and/or physiotherapy after surgery was not available for the sample and may have impacted physical and mental health and functioning outcomes.

## Conclusion

For elective surgical procedures, it is critical that candidates are both clinically appropriate and have a clear and informed preference for the procedure. The results highlight that patients who had spine surgery reported more SDM with their providers while patients who had TJR tended to be better informed about their procedures, identifying strengths in both spine and TJR surgery decision making. Future work is needed to determine the best way to deploy the measures in health systems to monitor and track improvements.

## Supplementary Information

**Additional file 1.**

**Additional file 2.**

## Data Availability

Investigators interested in the de-identified data will need to contact KS and show IRB approval for secondary use and execute a data use agreement. No information that contains identifiers or that could be used to link an individual to the data will be included in the de-identified data set. Study materials are available from the corresponding author. Analytic code is included in the supplemental materials.

## Abbreviations

TJR: Total joint replacement
SDM: Shared Decision Making
SDMP: Shared Decision Making Process
IPC: Informed, Patient-Centered
OA: Osteoarthritis
HD: Herniated Disc
SS: Spinal Stenosis
PROMs: Patient-Reported Outcome Measures
DQI: Decision Quality Instruments
GEE: Generalized Estimating Equations
