# Supplementary material for: Assessing the quality of shared decision making for elective orthopedic surgery across a large healthcare system: cross-sectional survey study
Source: BMC Musculoskelet Disord. 2021 Nov 19;22:967. doi: 10.1186/s12891-021-04853-x (PMC8605511; doi:10.1186/s12891-021-04853-x)
Supplement: Supplementary file 1 — Additional file 1. [file 12891_2021_4853_MOESM1_ESM.docx]

**Supplemental File**

**eTable 1:** Patient Eligibility Criteria

|  | *Eligible* | *Ineligible* |
| --- | --- | --- |
| Total joint arthroplasty | - Had a primary total hip or primary total knee replacement performed by participating surgeon - Had an equivalent diagnosis of hip or knee osteoarthritis - Between 12-26 weeks post-operative - Completed a patient reported outcomes measure assessment prior to their operation - Preferred language listed as English | - Had a severe cognitive impairment such that they could not consent for themselves - Hip patients with a hip fracture up to 12 months prior - Hip and knee patients with avascular necrosis up to 12 months prior |
| Spine | - Had spinal fusion, laminectomy, or discectomy performed by participating surgeon - Diagnosis of lumbar herniated disc or lumbar spinal stenosis - 4-26 weeks post-operative - Preferred language listed as English | - Had a severe cognitive impairment such that they could not consent for themselves - Spinal surgery patients with certain symptoms (e.g., bladder or bowel changes) that would indicate non-elective procedure |
